# Fifteen years of programme implementation for the elimination of Lymphatic Filariasis in Ghana: Impact of MDA on immunoparasitological indicators

**DOI:** 10.1371/journal.pntd.0005280

**Published:** 2017-03-23

**Authors:** Nana-Kwadwo Biritwum, Dziedzom K. de Souza, Benjamin Marfo, Samuel Odoom, Bright Alomatu, Odame Asiedu, Abednego Yeboah, Tei E. Hervie, Ernest O. Mensah, Paul Yikpotey, Joseph B. Koroma, David Molyneux, Moses J. Bockarie, John O. Gyapong

**Affiliations:** 1 National Neglected Tropical Diseases Programme, Ghana Health Service, Accra, Ghana; 2 Parasitology Department, Noguchi Memorial Institute for Medical Research, University of Ghana, Legon, Accra, Ghana; 3 FHI360, Ghana Office, Accra, Ghana; 4 Liverpool School of Tropical Medicine, Liverpool, United Kingdom; 5 European and Developing Countries Clinical Trials Partnership (EDCTP), Africa Office, Cape Town, South Africa; 6 University of Health and Allied Sciences, Ho, Ghana; McGill University, CANADA

## Background

Lymphatic filariasis (LF) is a disease found in the tropical and subtropical regions of the world, where it is a major public health problem. It is caused by the helminth parasites *Wuchereria bancrofti*, *Brugia malayi*, and *B*. *timori*, and is transmitted by mosquitoes. The availability of tools and strategies for the control of the disease [1] led to the World Health Assembly resolution (WHA 50.29) calling on member states to work towards the elimination of LF as a public health problem by 2020 [2]. The World Health Organization (WHO) launched the Global Programme to Eliminate Lymphatic Filariasis (GPELF) in 2000, with the principal objective of breaking the cycle of transmission of *W*. *bancrofti* and *Brugia spp*. through the application of annual mass drug administration (MDA) to entire at-risk populations for a period of five to six years. In 2012, 73 countries were endemic (81 at the onset of the GPELF), with 1.4 billion people at risk, 120 million people infected, and 40 million people affected by LF-related morbidity [3].

Ghana was one of the first countries to implement MDA, and the Ghana Filariasis Elimination Programme (GFEP) was established in June 2000. The strategy of annual MDA was based on the WHO guidelines for countries that are coendemic for LF and onchocerciasis [4]. This strategy recommends the use of ivermectin (IVM) and albendazole (ALB) treatment regimens to be undertaken annually for identified implementation units (IUs) where the antigen prevalence is above 1%. Monitoring the impact of MDA on transmission of LF in Ghana involved several impact assessment surveys, based on WHO guidelines and protocols [4,5]. In the implementation of these guidelines, various challenges were encountered, which have contributed to their revision. This paper presents an overview of the evolution of the national programme, describes the impact of MDA on the transmission of LF in Ghana, and discusses the challenges encountered in the process.

## Programme implementation

Prior to the implementation of the LF program, there were 110 districts in Ghana. A prevalence map developed in 2000 indicated that LF was endemic in 49 of 110 endemic districts [6]. LF endemic districts were designated as the IUs. Subsequently, the Ministry of Local Governments undertook several redemarcations of the administrative districts. Between 2003 and 2012 the number of IUs increased to 98 out of a total of 216 districts. The programme also underwent a gradual upscaling plan of MDA implementation from the initial five start-up districts in 2000 to all 74 districts in 2006 (Table 1).

**Table 1:** Upscaling plan of the Ghana Filariasis Elimination Programme indicating the number of implementation units by implementation year.

Redemarcations	2000–2001	2001–2002	2002–2003	2003–2004	2004–2005	2005–2006	Total Number of Districts
Prior to 2003	5	14	30	40	48	49	110
2003	6	18	38	51	60	61	138
2007	8	24	46	61	72	74	170
2012	10	29	57	74	95	98	216

This map guided the implementation of MDA by the GFEP, now part of the Neglected Tropical Disease programme. Baseline microfilaremia (MF) prevalence data were also collected from sentinel sites for impact monitoring. Following successful midterm impact assessments, pretransmission assessment surveys were undertaken after five to six years of treatment in the districts. From 2010, transmission assessment surveys (TAS) were undertaken in districts where sentinel and spot check surveys revealed MF prevalence to be below 1%. This was to allow for a decision to stop MDA in these districts. School children aged six to seven years were sampled using the TAS protocol [5].

## Programme outcomes

**Fig 1:**
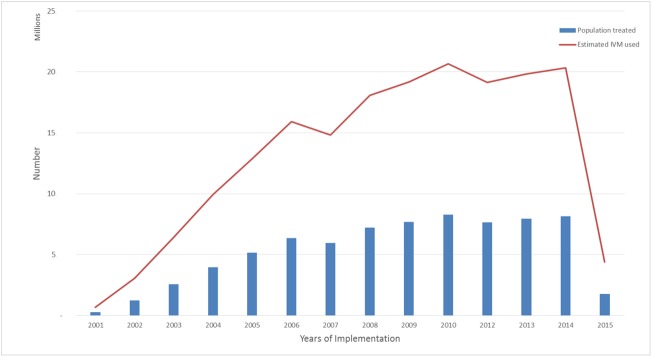
Number of people treated and ivermectin (IVM) tablets distributed from 2000–2015.

During the last 15 years of the programme, a cumulative total of over 74 million people were treated in the 98 endemic districts across the country. An estimated 185 million IVM tablets and 74 million ALB tablets were distributed within the period. The number of people treated peaked in 2010 as a result of the upscaling and efforts to improve treatment coverage. However, this decreased in 2015 as 76 endemic districts passed TAS and treatment stopped in such districts (Fig 1). It must be noted that while the population of Ghana is estimated at 27 million, less than half of these actually live in endemic districts, with a majority living in urban areas where MDA faces several challenges. Fig 1 also shows the number of people treated in each year, and how the amount increased with the upscaling of the programme.

Baseline data collected in 2000 before the first annual MDA in the programme’s five start-up districts (Awutu-Efutu-Senya [AES], Ahanta West, Builsa, Kassena Nankana, and Sissala) revealed MF prevalence to be between 19.8% and 29.6% and immunochromatographic test (ICT) prevalence to be between 33.1% and 45.4%. Between 2001 and 2009, yearly surveillance and upscaling of implementation activities have been undertaken in different districts in the country (S1–S6 Tables). The 2003 parasitological surveys provided an opportunity to conduct midterm impact assessments in the five start-up districts of the programme, and collect baseline data for a selection of districts that were being recruited into the programme for the first time. In 2007, night blood surveys were undertaken as part of a cross-sectional study in the five start-up districts. Two sentinel sites and one cross-check site were surveyed in each district, with a sample size of 500 individuals targeted for the adult surveys.

**Fig 2:**
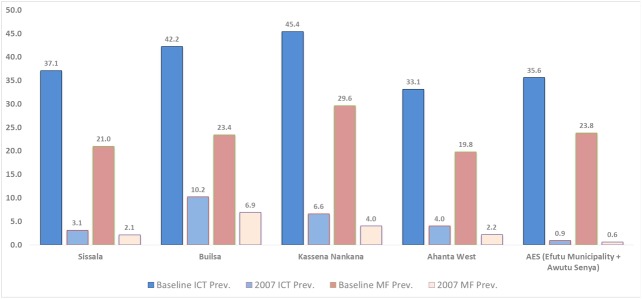
Summary results of baseline and 2007 ICT and microfilaremia (MF) prevalence in the five start-up districts.

The MF prevalence by district ranged from 0.6%–6.9% (Fig 2). The district MF prevalence was more than 1% for all the districts except the AES district (0.6%). The minimum parasite counts were lowest (1 mf/ml of blood) in AES and highest in Kassena Nankana district (588 mf/ml of blood). Generally, downward trends were observed across the five start-up districts with significant reduction in MF prevalence from the beginning of the programme. In AES, for example, prevalence levels declined from 45.7% at baseline to 2.9% at midterm, then 0.9% after year six. Similar downward trends could be demonstrated from other implementing districts. However, a number of communities in the districts with more than seven rounds of MDA had an MF prevalence that remained above the 1% level recommended for interruption of transmission. At this time point in the program, TAS procedures were not properly established and as such it was difficult to determine whether MDA should be stopped in the districts where prevalence was higher than 1% in some communities. Subsequent implementation of TAS 1 surveys between 2010 and 2015 showed that 76 districts had antigen prevalence below 1%. Consequently, treatment stopped in these districts.

## Programme challenges

Generally, MF prevalence and immunoparasitologic indicators have shown significant and continual downward trends in all districts since the inception of the programme. Many districts were able to reduce their MF prevalence to less than 1% and had undertaken TAS. However, the recommended five to six years required to break transmission needs to be reviewed, as 22 districts with over ten years of MDA and coverage above 65% (71.6%–88.4%) still have prevalence above the recommended 1% level. The number of years required to interrupt transmission appears to depend on the MF prevalence at the inception. It is important to note that several factors, in particular therapeutic coverage and adherence rates, geographical coverage [7,8], mosquito vector characteristics [9], and drug regimens [10], may all influence the rate of decline of parasitological parameters, which measure the success of the programme, will need to be considered in the process of monitoring and evaluation of MDA programmes.

In implementing the immune-parasitological surveys, several challenges were encountered, despite the availability of operational guidelines. The operational guidelines provide the best practices, some of which are not always applicable in every setting or need to be adapted to specific situations. These are discussed below.

### Timing of surveys

Serious technical challenges due to poor timing of surveys and the ICT card quality were encountered. For example, the 2001 survey was undertaken during the cold, dry, dusty harmattan season (December to February). Although samples were collected for both blood smears and antigen tests, the slides were heavily contaminated with dust particles, making processing and reading of the slides impossible, and so the results had to be abandoned. Vasoconstriction due to the cold weather made bleeding of individuals for the survey also difficult, particularly during night surveys between 10 p.m. and 2 a.m. Furthermore, the ICT cards required reading of results within ten minutes. However, samples were collected and kept overnight due to poor visibility and lighting, leading to a large number of false positive readings. The results obtained for both the ICT and blood slides for MF (S1 Table) therefore could not be applied for programmatic decision-making. Because these activities were undertaken in the districts for the first time, any interactions with locally based personnel would not have prevented the inadequate timing, as it was impossible to determine the extent to which the weather pattern would influence the results. Thus, for subsequent surveys the timing of the monitoring activities, together with logistical challenges, influenced the number of sites that could be visited for any particular annual survey.

### Availability of ICT cards

In several surveys, the use of ICT cards was limited by their availability, coupled with technical challenges, such as the cold chain requirement and varied field performance due to the short reading time frame. Being at the early stages of implementation, the Ghana program had challenges procuring enough ICT cards through its donors due to funding limitations. For example, in the 2002 surveys, the ICT card for antigenemia were only used in Agona and Nzema East districts due to the limited availability. Thus, the value of antigen tests in programmatic decision-making, especially at the early stages of the programme, was greatly reduced, as prevalence results were either based on ICT cards or microscopy. This did not enable an effective comparison of the results obtained from the different districts in order to determine which districts were to be enrolled into the MDA program. Furthermore, the use of different tools, i.e., antigen versus microscopy, may result in data differences [11], as observed in the programme implementation. We would therefore recommend that control programmes strive to consistently use the same tools throughout their evaluations in order to appropriately assess the success of their activities. Furthermore, the development of a new diagnostic, the Alere LF Test Strip, which is less expensive and more stable than the ICT card and does not require a cold chain [12], dramatically improved surveillance activities.

### Inadequate sample sizes

Another challenge encountered in the early years of the programme implementation was inadequate sample sizes. In some communities, sample sizes as low as 22 individuals were used to assess prevalence levels, leading to disparities in data from one year to the next. While the differences between results may be related to the use of antigen versus MF, they may also be a result of sample size and selection bias [11]. Thus, lower prevalence was obtained in such cases than when the appropriate sample size was used. For example, it was noted that for each of the five start-up districts, with the exception of Sissala, there was an increase in prevalence from the fifth-year assessments to the post-year six assessments. This was because efforts were made to improve on the sample sizes employed for the surveys, thus providing a more representative picture of the true MF prevalence within these districts by increasing the power of the surveys. In some communities, the required samples sizes were not met and the program extended the sampling to neighbouring communities so that these could be achieved. An example was in Nadowli, which had a prevalence of 4% and 17% at baseline and midterm evaluations, respectively. Limitations of LF programmes in terms of inadequate or small sample sizes have been reported [13]. Thus, the need for achieving the appropriate sample sizes in evaluating programmes cannot be overemphasized.

### Funding and logistic challenges

The availability of funding influenced the upscaling of the programme and the number of districts that could be evaluated. However, MF prevalence surveys were undertaken in spite of funding and logistical challenges. As such, modifications were made to the recommended WHO methods for monitoring and evaluation, so as to match funding and logistic availability. At the beginning of the programme, the WHO guidelines were followed requiring 200 samples per site and two sentinel and one cross-check site per district. This worked well in the early days when the program only had few districts to survey. However, during the upscaling phases, sampling many districts based on these recommendations proved challenging as a result of the increased workload, with considerable financial and logistic challenges. As such, fewer sites were selected to represent wider geographic areas. As another example of a modification made during the program, where sample sizes were not met in particular sites selected for evaluation, the surveys were extended to neighbouring communities so as to meet the recommended sample sizes. This enabled the program to have a more reliable estimate of LF prevalence. In most cases, the data obtained from these modifications led to the decision to undertake TAS and stop MDA. However, in some cases, additional data needed to be collected for more reliable estimates. The availability of funding determines the ability to undertake surveys [14]. It is hoped that the financial commitments by international partners such as United States Agency for International Development (USAID), Department for International Development (DFID), World Bank, the Bill & Melinda Gates Foundation following the London Declaration (2012), and the subsequent meeting in Paris (2014) will see major improvements in the funding needs of control programmes [15,16].

## Outlook for the next five years

The Ghana LF programme has made significant progress towards the 2020 elimination goal. However, the next five years will be crucial if the target is to be achieved. As of 2015, there are 22 districts defined as “hotspots” that have had at least ten years of MDA but with MF prevalence still above the recommended 1% level. It is therefore important to investigate these districts and intensify MDA to address the challenges identified. In addition, vector control has been shown to greatly impact the transmission of LF [17,18], and as such, it will be important to complement MDA in these districts, with vector control strategies.

Furthermore, a recent study using the WHO data quality assessment (DQA) tool revealed inaccuracies of coverage results reported at the service delivery points [8], pointing to challenges in the data management and reporting tools of the programme. Therapeutic coverage and adherence to MDA are two important indices used to assess the success of MDA campaigns [19,20].

The WHO’s guidelines for confirming the interruption of LF transmission require mapping to identify LF-endemic areas; conducting four to six rounds of yearly MDA in endemic areas with at least 65% therapeutic coverage; and undertaking monitoring and evaluation (pre-TAS), followed by TAS to determine when the MDA can be stopped. Currently, 76 endemic districts have undertaken TAS 1. However, for these districts, it is important to undertake a further TAS 2 as required by WHO [5]. Furthermore, there is the need to provide evidence of absence of transmission in the mosquito vectors of LF [3]. As such, while the programme is intensifying its efforts in “hotspot” districts, it is also planning and executing TAS 2 in addition to entomological investigations [21] in districts where MDA has stopped.

As in many other countries, districts with less than 1% ICT prevalence were considered nonendemic for LF and as such were not treated by MDA. However, LF is a focal disease and the 50-km grid method [6], the WHO LF mapping protocol [4], and TAS [5] have the potential to miss LF endemic communities [21,22]. It is therefore important to develop new LF mapping protocols that could be used to reevaluate districts previously considered nonendemic in LF endemic countries. In Ghana, new LF cases have been reported in some districts considered nonendemic. It is therefore crucial to determine the extent of LF endemicity in these districts and if endemic there will be the need to complement MDA with vector control so as to meet the 2020 target goals.

It has been argued that the financial needs of LF control programmes would reduce as the activities are scaled down towards the end of the programme. However, the end of the programmes would rather call for TAS and entomological surveys that require financial resources. While pool screening and other innovative approaches [23] are being developed to reduce the cost associated with entomological surveys, it is important that funding be sustained or even increased to sustain the surveys that will demonstrate elimination of LF.

In conclusion, the immunoparasitological surveys (midterm impact assessment and pre-TAS) conducted as part of the GFEP have revealed the impact of MDA in reducing the prevalence of LF in many districts, many of which have reached the post-MDA implementation phase. However, these surveys have also highlighted the need to review the five to six years of recommended MDA required to interrupt transmission, as persistent transmission has been observed in some communities. The DQA in initial districts [8] revealed some inaccuracies in reported coverage, and the program is working towards improving the data reported in the remaining districts through training on the effective use of the data reporting tools and processes. While the challenges of persistent transmission are yet to be resolved, the distribution of insecticide-treated bed nets could accelerate the elimination of LF in these areas as high coverage of bed nets should reduce the transmission of the disease [17,18]. It is hoped that, for the few countries that have yet to implement MDA or are still at the beginning of their programmes, the information provided herein will serve as additional guidance towards achieving successful elimination. The adaptability of operational guidelines to country settings is therefore important in achieving a successful programme.

## Supporting information

S1 Table 2001 night blood survey results showing antigen prevalence using ICT cards. (DOCX)

S2 Table 2002 night blood survey results showing antigen and MF prevalence. (DOCX)

S3 Table 2003 immunoparasitologic survey results showing antigen and MF prevalence. (DOCX)

S4 Table 2004 blood surveys results showing antigen and MF prevalence. (DOCX)

S5 Table 2005 summary night blood survey results showing antigen and MF prevalence and antigen prevalence among children under five years. (DOCX)

S6 Table Results of 2009 blood surveys. (DOCX)
