# Supplementary material for: Fifteen years of programme implementation for the elimination of Lymphatic Filariasis in Ghana: Impact of MDA on immunoparasitological indicators
Source: PLoS Negl Trop Dis. 2017 Mar 23;11(3):e0005280. doi: 10.1371/journal.pntd.0005280 (PMC5363798; doi:10.1371/journal.pntd.0005280)
Supplement: S1 Table — (DOCX) [file pntd.0005280.s001.docx]

Supplementary Table 1: 2001 Night Blood Survey Results showing Antigen Prevalence using ICT Cards

| 2001 ICT Card Test Results | | | |
| --- | --- | --- | --- |
| DISTRICT | No. Tested | ICT Positive | ICT Prevalence (%) |
| Wa | 200 | 169 | 85 |
| Lawra | 175 | 129 | 74 |
| Jirapa | 200 | 118 | 59 |
| Nadowli | 200 | 163 | 82 |
| Sissala | 200 | 155 | 78 |
| Kassena Nankana | 200 | 169 | 85 |
| Builsa | 200 | 156 | 78 |
| Bongo | 200 | 146 | 73 |
| Bolga | 200 | 141 | 71 |
| Total | 1775 | 1346 | 76 |
