# Supplementary material for: Fifteen years of programme implementation for the elimination of Lymphatic Filariasis in Ghana: Impact of MDA on immunoparasitological indicators
Source: PLoS Negl Trop Dis. 2017 Mar 23;11(3):e0005280. doi: 10.1371/journal.pntd.0005280 (PMC5363798; doi:10.1371/journal.pntd.0005280)
Supplement: S2 Table — (DOCX) [file pntd.0005280.s002.docx]

Supplementary Table 2: 2002 Night Blood Survey Results showing Antigen and Microfilaraemia Prevalence

| Region | District | Number Positive (N) | | People Examined | Prevalence (%) | |
| --- | --- | --- | --- | --- | --- | --- |
|  |  | MF | ICT |  | ICT | MF |
| Upper East | Bawku East | 21 | - | 505 | - | 4.2 |
| Northern | East Mamprusi | 1 | - | 230 | - | 0.4 |
| Northern | West Mamprusi | 5 | - | 714 | - | 0.7 |
| Central | Agona | 10 | 32 | 136 | 23.5 | 7.4 |
| Western | Nzema East | 0 | 27 | 199 | 13.6 | 0.0 |
| Total | 5 | 37 | 59 | 1784 | 3.3 | 2.1 |
