# Supplementary material for: Fifteen years of programme implementation for the elimination of Lymphatic Filariasis in Ghana: Impact of MDA on immunoparasitological indicators
Source: PLoS Negl Trop Dis. 2017 Mar 23;11(3):e0005280. doi: 10.1371/journal.pntd.0005280 (PMC5363798; doi:10.1371/journal.pntd.0005280)
Supplement: S3 Table — (DOCX) [file pntd.0005280.s003.docx]

Supplementary Table 3: 2003 Immuno-parasitologic Survey Results showing Antigen and Microfilaraemia Prevalence

| REGION | Districts | No. Sampled | Results | | Prevalence (%) | |
| --- | --- | --- | --- | --- | --- | --- |
|  |  |  | ICT Positive | MF Positive | ICT | MF |
| Upper West | 5 | 415 | 14 | 33 | 14 | 8.0 |
| Upper East | 4 | 353 | 39 | 53 | 39 | 15.0 |
| Northern | 1 | 486 | 3 | 0 | 3 | 0.0 |
| Central | 3 | 603 | 11 | 1 | 11 | 0.2 |
| Western | 2 | 657 | 28 | 11 | 5 | 4.3 |
| Total | 15 | 2514 | 95/1643 | 98/2514 | 5.8% | 3.9% |
