# Supplementary material for: Fifteen years of programme implementation for the elimination of Lymphatic Filariasis in Ghana: Impact of MDA on immunoparasitological indicators
Source: PLoS Negl Trop Dis. 2017 Mar 23;11(3):e0005280. doi: 10.1371/journal.pntd.0005280 (PMC5363798; doi:10.1371/journal.pntd.0005280)
Supplement: S4 Table — (DOCX) [file pntd.0005280.s004.docx]

Supplementary Table 4: 2004 Blood Surveys Results showing Antigen and Microfilaraemia Prevalence

| REGION | Districts | Communities | RESULTS | | | | |
| --- | --- | --- | --- | --- | --- | --- | --- |
|  |  |  | No. Sampled | MF positive | MF Prevalence (%) | ICT Positive | ICT Prevalence (%) |
| Upper West | 4 | 8 | 729 | 117 | 16.0 | 146 | 20.0 |
| Upper East | 2 | 4 | 156 | 19 | 12.2 | 44 | 28.2 |
| Northern | 1 | 3 | 516 | 17 | 3.3 | 103 | 21.6 |
| Brong Ahafo | 1 | 4 | 530 | 15 | 2.8 | 50 | 35.5 |
| Eastern | 1 | 3 | 519 | 5 | 1.0 | 5 | 1.8 |
| Greater Accra | 1 | 3 | 483 | 1 | 0.2 | 38 | 7.9 |
| Total | 10 | 25 | 2933 | 174 | 5.9 | 386 | 17.5 |
