# Supplementary material for: Fifteen years of programme implementation for the elimination of Lymphatic Filariasis in Ghana: Impact of MDA on immunoparasitological indicators
Source: PLoS Negl Trop Dis. 2017 Mar 23;11(3):e0005280. doi: 10.1371/journal.pntd.0005280 (PMC5363798; doi:10.1371/journal.pntd.0005280)
Supplement: S5 Table — (DOCX) [file pntd.0005280.s005.docx]

Supplementary Table 5: 2005 Summary Night Blood Survey Results showing Antigen and Microfilaraemia Prevalence and Antigen Prevalence among Children under Five Years

| REGION | Districts | Communities | Microfilaremia | | ICT Antigen | |
| --- | --- | --- | --- | --- | --- | --- |
|  |  |  | Sampled | MF Prevalence (%) | ICT Prevalence (%) | U-5 ICT Prevalence. |
| Western | Ahanta West | 3 | 502 | 3.2 | 15.5 | 3.9 |
|  | Nzema East | 3 | 371 | 0.0 | 2.0 | 0.0 |
| Central | A.E.S | 4 | 500 | 0.2 | 6.2 | 0.0 |
|  | Agona | 4 | 465 | 1.3 | 10.9 | 4.2 |
| Northern | East Mamprusi | 3 | 474 | 0.0 | 0.4 | - |
|  | West Mamprusi | 3 | 505 | 1.2 | 5.5 | - |
| UWR | K.N.D | 4 | 505 | 4.0 | 7.1 | 3.8 |
|  | Builsa | 3 | 500 | 3.6 | 11.2 | 2.3 |
| UER | Nadowli | 3 | 500 | 6.4 | 7.0 | 3.3 |
|  | Sissala | 4 | 500 | 0.0 | 1.5 | 0.0 |
| Total | 10 | 34 | 4822 | 2.1 | 6.8 | 2.6 |
