# Supplementary material for: Fifteen years of programme implementation for the elimination of Lymphatic Filariasis in Ghana: Impact of MDA on immunoparasitological indicators
Source: PLoS Negl Trop Dis. 2017 Mar 23;11(3):e0005280. doi: 10.1371/journal.pntd.0005280 (PMC5363798; doi:10.1371/journal.pntd.0005280)
Supplement: S6 Table — (DOCX) [file pntd.0005280.s006.docx]

Supplementary Table 6: Results of 2009 Blood Surveys

| **Districts** | **No. of communities** | **No. Sampled** | **MF Positive** | **Prevalence (%)** | **Parasite Count** | **Density** |
| --- | --- | --- | --- | --- | --- | --- |
| Agona | 10 | 1199 | 0 | 0.0 | 0 | 0 |
| Tarkwa Nsuaem | 5 | 664 | 1 | 0.2 | 34 | 567.8 |
| Axim Municipal | 7 | 596 | 17 | 2.9 | 891 | 875.276 |
| Lawra | 9 | 1074 | 81 | 7.5 | 2440 | 503.062 |
| Nadowli | 6 | 969 | 20 | 2.1 | 429 | 358.215 |
| Wa | 9 | 1228 | 101 | 8.2 | 2494 | 412.374 |
| Jirapa | 7 | 1325 | 33 | 2.5 | 452 | 228.739 |
| Bolgatanga | 9 | 1037 | 31 | 3.0 | 301 | 162.152 |
| Bongo | 8 | 1007 | 34 | 3.4 | 307 | 150.791 |
| Bawku West | 6 | 978 | 14 | 1.4 | 643 | 767.007 |
| West Mamprusi | 8 | 1160 | 7 | 0.6 | 300 | 715.714 |
| Tolon Kumbungu | 9 | 1025 | 2 | 0.2 | 11 | 91.85 |
| Savelugu Nanton | 8 | 954 | 0 | 0.0 | 0 | 0 |
| Zabzugu Tatale | 6 | 956 | 0 | 0.0 | 0 | 0 |
| Nanumba | 6 | 1003 | 1 | 0.1 | 2 | 33.4 |
| Total | 113 | 15175 | 342 | 2.3 | 8304 | 405.488 |
